# Role of Allium cepa and Allium sativum extracts in oxidative stress, sperm parameters and histological abnormalities induced by deep-frying oil in testis

**DOI:** 10.1038/s41598-025-01826-1

**Published:** 2025-07-23

**Authors:** Mohamed Anwer Mohamed, Safa Hamdy Alkalash, Samah Baleegh Meawad, Mohamed Nafea Azab, Marwa A. Ahmed, Ahmad Gadalla, Muhammed Abdelbaeth Hassan Elfiky, Abdel Rahman Z. Abdel Rahman, Hatem Ali Ahmed Abdelmottaleb, Ayman Sabry Yassin Al Sayed, Wafaa Yahia Alghonemy, Amira Osman, Abeer A. Almowafy, Ahmed Mohamed GadAllah

**Affiliations:** 1 Department of Forensic Medicine and Clinical Toxicology, Faculty of Medicine, Al Azhar University, Assiut, Egypt; 2 Department of Community Medicine and Healthcare, Faculty of Medicine, Umm Al-Qura University, Al-Qunfudha, Saudi Arabia; 3 Department of Forensic Medicine and Clinical Toxicology, Faculty of Medicine for Girls, Al Azhar University, Cairo, Egypt; 4 Department of Physiology, Faculty of Medicine, Al Azhar University, Assiut, Egypt; 5 Department of Medical Microbiology and Immunology, Faculty of Medicine, Al Azhar University, Assiut, Egypt; 6 Department of Oral Biology, Faculty of Dentistry, Tanta University, Tanta, Egypt; 7 Department of Histology and Cell Biology, Faculty of Medicine, Kafrelsheikh University, Kafrelsheikh, Egypt; 8 International Islamic Institute for Population Studies and Research, Al-Azhar University, Cairo, Egypt; 9 Department of Food Sciences and Nutrition, College of Science, University of Bisha, P.O. Box 551, Bisha, 61922 Saudi Arabia

**Keywords:** AcL, AsL, Palm oil, Testes, Oxidative stress, Occupational health, Public health

## Abstract

Palm oil is commonly used for deep-frying street food preparation. Extremely vulnerable to oxidative stress and free radicals is testicular tissue. Antioxidant supplements are advised for reducing oxidative stress, boosting spermatogenesis, and neutralizing free radicals. The study evaluated the beneficial role of Allium cepa L(AcL) and Allium sativum L (AsL) extracts in oxidative stress, histopathological, and sperm parameters abnormalities induced by deep-frying palm olein oil in rat testis. Approximately 5 kg of falafel dough tablets were daily deep-fried in oil. For three days, the frying procedure required six hours per day. The animals received daily treatment for four weeks after being split into equal groups (control, AcL, AsL, fresh palm oil (FPO), single heated palm oil (SHPO), repeated heated palm oil (RHPO), AcL + RHPO, AsL + RHPO, and AcL + AsL + RHPO). Serum testosterone level, testicular tissue of malondialdehyde (MDA), glutathione (GSH), superoxide dismutase (SOD), glutathione peroxidase (GSH-Px), and sperm parameters were measured. In addition, a histopathological examination of the testis was performed. Repeatedly heating oil increased considerably the MDA alongside significant depletion of GSH, SOD, and GSH-Px. Serum levels of testosterone and sperm parameters were decreased significantly with testicular damage. Considerable improvements in oxidative stress, sperm parameters, and testicular histology were observed with concomitant administration of AcL and AsL. SHPO showed no significant changes. In the testes of rats subjected to oxidative stress caused by RHPO, extracts of AcL and AsL exhibit strong antioxidant protection. The study concluded that AcL and AsL extracts have potent antioxidant protection in the testes of rats exposed to RHPO-induced oxidative stress.

## Introduction

Street foods are foods and drinks that are readily consumable without requiring any processing or preparation and are offered for sale on the street and in other public areas^1^. The development of urbanization and globalisation has caused a change in eating habits during the past few decades^2^.

As a result of a lot of time expended outdoors, women work, and insufficient time for cooking, fried street food may be the cheapest and most accessible way to nutrition outside the home; in addition, fat and oil give a unique flavor and palatability, all of these, increases the fried food or fast-food consumption in our society.

Because of its simplicity and unique effects on food flavour and texture, deep fat frying—which includes submerging food in hot oil at temperatures between 140 and 200 degrees Celsius—is a popular cooking technique^3,4^. During this process, the food absorbs a small amount of oil, which causes the food to fry and produce a particular number of breakdown products from the oil^5^. Numerous processes, including oxidation, hydrolysis, isomerisation, and polymerisation, occur during deep-frying, changing the flavour and degrading the chemicals in cooking oil, impacting the oil’s quality and fried foods^6^.

One of the most widely used oils in the world is palm oil. Nigeria, Indonesia, Malaysia, Thailand, and Colombia are the top palm oil producers^7^. Saturated fatty acids are far more prevalent in palm oil. 10.6% linoleic acid, 42.1% oleic acid, and 38.3% palmitic acid are found in palm olein. It is more stable than vegetable oils with high levels of polyunsaturated fatty acids. Therefore, using this oil to make convenient and practical food makes sense. Nonetheless, the primary cause of palm oil’s numerous uses and varied applications is its comparatively inexpensive cost^8,9^.

In addition to their culinary uses, AcL and AsL have drawn much interest due to their practical health advantages. AcL and AsL have mainly been credited with the following functional health benefits: (1) modifying risk factors for cancer, diabetes, and cardiovascular diseases; (2) boosting immune function; (3) having antimicrobial and antischistosomal effects; (4) improving xenobiotic detoxification; (5) hepatoprotection; and (6) having antioxidant effects^10–13^.

To the best of our knowledge, our previous research^14^ was the first study in the literature to discuss the effect of repeatedly heated palm olein oil on the testes of rats. The current study assessed how AcL and AsL extracts affected oxidative stress, sperm parameters, and histological abnormalities in rat testis caused by repeatedly heating palm oil.

## Materials and methods

### Experimental animals

For the investigation, 72 mature male Wistar rats weighing 160 and 200 g were employed. The rats were purchased from Assiut University’s animal house in Egypt. The experimental protocol was authorized by the Faculty of Medicine Ethics Committee of Al-Azhar University in Egypt, and it was conducted following the National Institutes of Health Guide for the Care and Use of Laboratory Animals in accordance with ARRIVE guidelines. Eight rats were kept in metabolic cages with a 12-hour light/dark cycle, a temperature of 22 ± 2ºC, a relative humidity of 50 ± 5%, and unrestricted access to fresh water and rat chow, all following standard laboratory animal care standards. Before the trial started, the animals could acclimate for seven days.

### Preparation of repeatedly heated refined palm Olein oil (RHPO)

In Assiut, Egypt, refined palm oil was bought from a neighborhood market. One liter of oil was extracted and labeled “fresh palm oil” (FPO). A modified technique outlined by Chao et al.^15^ and our earlier investigation^14^ was used to manufacture RHPO. Six kilograms of FPO were added to a stainless steel wok and heated to 180 degrees Celsius. Every day, roughly 5 kg of falafel dough tablets were fried in oil. It took six hours every day to fry. One litre of oil was extracted from this sample and designated as single heated palm oil (SHPO). Oil heated three times (RHPO) was obtained by repeating the same procedure. Following the daily frying procedures, no extra new oil was added to compensate for the loss caused by the absorption of the fried falafel.

### Preparation of acl (onion) and AsL (garlic) extracts

We purchased fresh garlic and onion bulbs from the Al-Azhar University Faculty of Pharmacy’s therapeutic plant farm in Assiut, Egypt. The Department of Pharmacognosy verified their botanical identity and authenticity. Types of municipal garlic and red onions were chosen. The bulbs were prepared with care. Extracts from onions and garlic were made using the technique outlined by Ola-Mudathir et al.^16^. In a mixing machine, 100 g of onion and/or garlic were added and crushed with 100 ml of cooled, distilled water. After squeezing and filtering the resulting slurry through a fine cloth, the filtrate was promptly refrigerated until it was needed.

### Experimental design

After acclimatization, the rats were split into nine equal groups, each containing eight rats. The following course of treatment was employed:

Control group: received distilled water (1 ml/100 g BW/day). AcL group: received AcL (1 ml/100 g BW/day). AsL group: received AsL (1 ml/100 g BW/day). FPO group: received FPO (1 ml/100 g BW/day). SHPO group: received SHPO (1 ml/100 g BW/day). RHPO group: received RHPO (1 ml/100 g BW/day). AcL + RHPO group: received AcL (1 ml/100 g BW/day) followed by RHPO (1 ml/100 g BW/day), two h interval. AsL + RHPO group: received AsL (1 ml/100 g BW/day) followed by RHPO (1 ml/100 g BW/day), two h interval. AcL + AsL + RHPO group: received AcL (1 ml/100 g BW/day) and AsL (1 ml/100 g BW/day) followed by RHPO (1 ml/100 g BW/day), two h interval. The tested samples were orally by gavage for 28 days.

Weighing the rats was done at the start of the experiment and then every week until the final body weight was established. At the end of the study, After a 12-hour overnight fast after the 4-week research, the animals didn’t eat, then anesthetized with sodium pentobarbital (200 mg/kg IP) before cervical dislocation^17,18^.

### Collection and Preparation of tissue

The heart’s blood was drawn, placed in plain test tubes, and left to coagulate. Following a 15-minute centrifugation at 3000 g to separate the serum from the clotted blood samples, the serum was kept at −20ºC until the serum testosterone level was determined. Each rat’s left testis was promptly taken out, divided into two parts, and weighed with an electronic Shimadzu balance (BL-220 H; Kyoto, Japan) to the closest milligram. One part was blocked for histological analysis, while the other was utilized for biochemical testing (lipid peroxidation and antioxidant enzymes).

Testicular tissues were perfused with a pH 7.4 PBS (phosphate buffered saline) solution with 0.16 mg/ml heparin to eliminate red blood cells and clots. Then, using a Potter-Elvehjem type homogenizer, they were homogenized (10% w/v) in ice-cold potassium phosphate buffer at pH 7.4 that contained one mM EDTA. Supernatants were extracted for biochemical examination after the homogenate was centrifuged at 4000 g for 15 min at 4ºC.

### Biochemical analysis

Superoxide dismutase (SOD), reduced glutathione (GSH) level, glutathione peroxidase (GSH-Px) activity, and lipid peroxidation (malondialdehyde) were measured in accordance with Ohkawa et al.^19^, Beutler et al.^20^, Chiu et al.^21^, and Nishikimi et al.^22^ respectively. Following the manufacturer’s instructions, enzyme-linked immunosorbent assay (ELISA) kits were used to assess the serum level of testosterone.

### Epididymal sperm count, motility, and morphology

The epididymal sperm count was determined using the Linder et al. method^23^ and Luo et al.^24^. Therefore, the epididymis was sliced using anatomical scissors in 5 mL of physiological saline and incubated for 2 min at 32 ^o^C to collect epididymal spermatozoa. An aliquot of this solution was added to Malassez cells, and the motile sperm were counted using a 400x microscope magnification. The total amount of sperm was then counted after the non-motile sperm counts. Sperm motility was represented by the proportion of motile sperm to all sperm counted.

With a slight modification (a drop of the sperm suspension was deposited onto a fresh glass plate for each sample and left to dry in the air), the proportion of morphologically aberrant spermatozoa was estimated as described by Terpsidis et al.^25^.

### Histopathological examination

Fresh testicular tissues were preserved in Bouin’s solution for histological analysis under a light microscope. After being fixed for two days, the samples were cleaned, dehydrated using a succession of ethanol grades, and then embedded in paraffin wax. A rotary microtome was used to create blocks and segment them at a 4 mm thickness. Sections were examined under a light microscope after being rehydrated in distilled water and stained with hematoxylin-eosin.

### Statistical analysis

All results are expressed as the mean ± SD. The normality of the data distribution was assessed using the Shapiro-Wilk and Kolmogorov-Smirnov tests. The data was analyzed using Turkey’s post hoc test and one-way analysis of variance (ANOVA). Graph Pad Software Inc., USA, version 7, was used for the statistical analysis. Statistical significance was established at p˂0.05.

## Results

### Animal observation, body weight, and weight of the testes

**Fig. 1:**
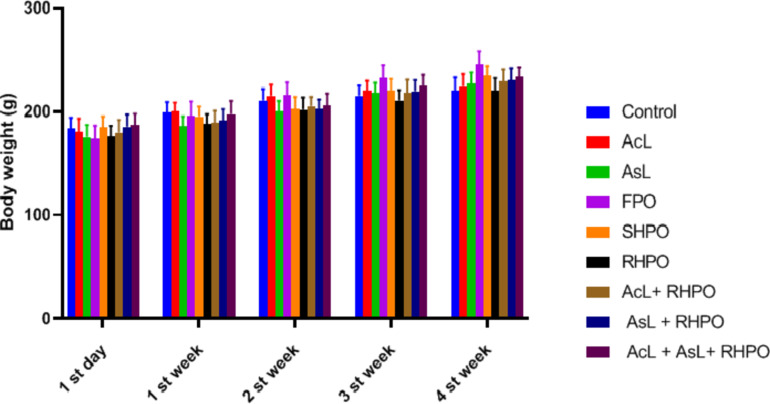
Effects of AcL and AsL extracts on testicular weight in rats given RHPO as opposed to the control group. With *n* = 8 in each group, the bars represent means ± SD. There is no statistically significant difference between the experimental groups.

No deaths or aberrant signs were seen after 4 weeks of the trial period. As seen in Fig. 1, throughout the experiment’s first, second, third, and fourth weeks, no discernible changes in the body weight of any of the tested groups were found when compared to the control group. The FPO group’s body weight has somewhat increased, but not noticeably.

**Fig. 2:**
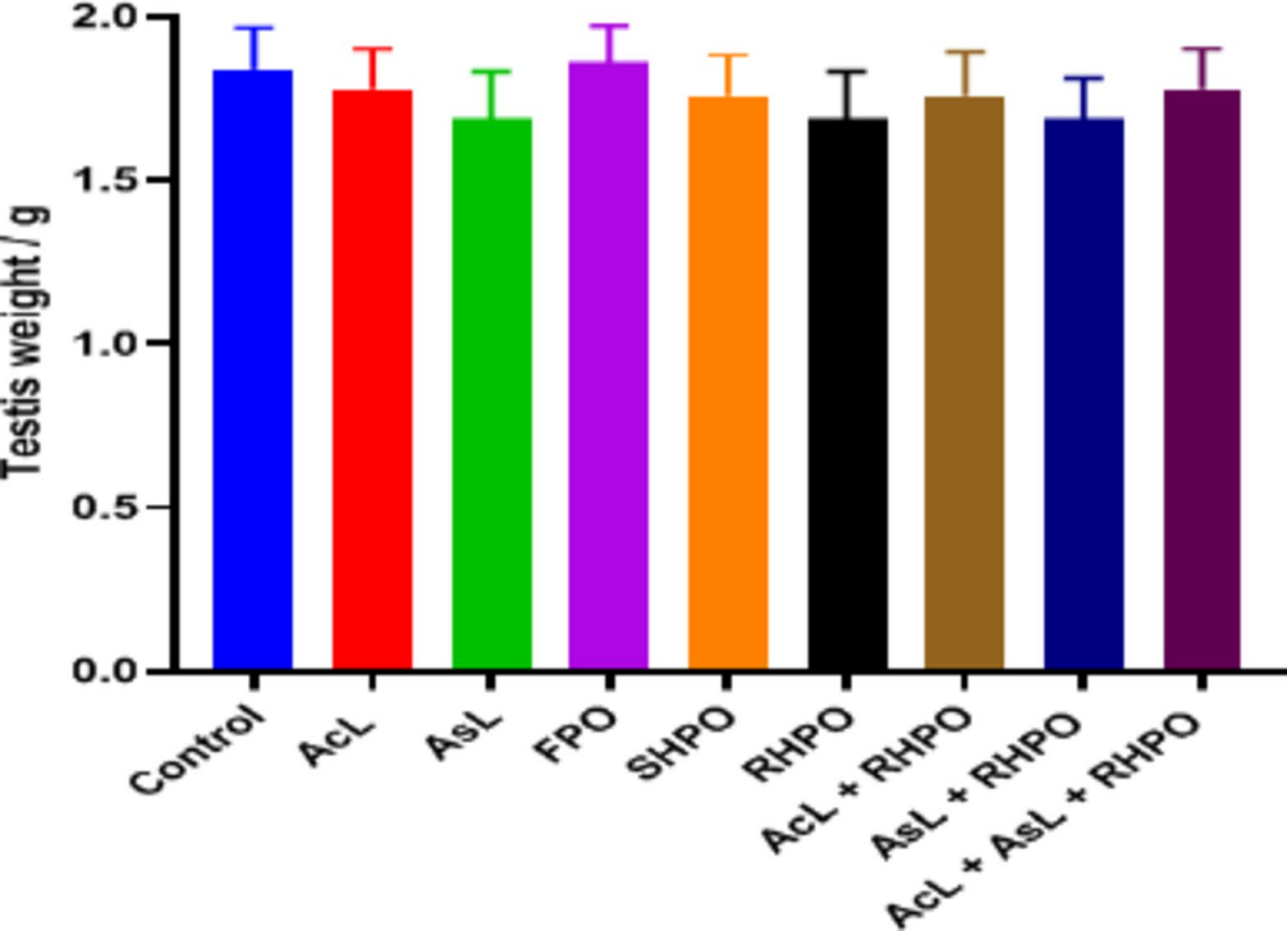
Effects of AcL and AsL extracts on body weight in rats given RHPO as opposed to the control group. With *n* = 8 in each group, the bars represent means ± SD. There is no statistically significant difference between the experimental groups.

Fig. 2 shows no discernible differences between the testicular weights of the tested and control groups.

### Biochemical parameters

#### Lipid peroxidation and antioxidant enzyme levels

**Fig. 3:**
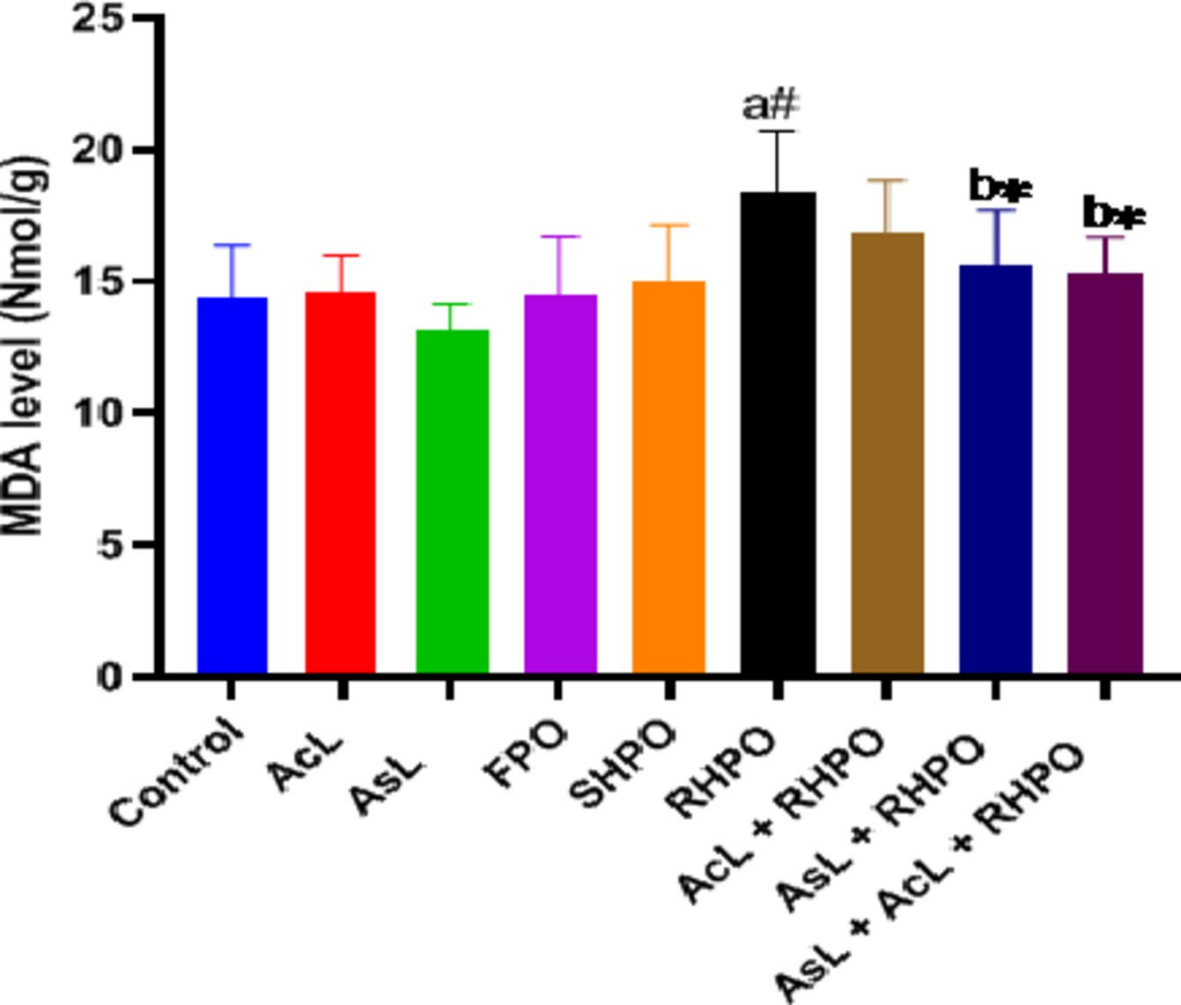
Effects of AcL and AsL extracts on the amount of testicular lipid peroxidation in rats given RHPO. With *n* = 8 in each group, the bars represent means ± SD. One-way ANOVA was used to analyze the data, followed by Turkey’s post hoc test. *p˂0.05 and #*p*˂0.01. a = control vs. AcL, AsL, FPO, SHPO, and RHPO. b = RHPO vs. AcL + RHPO, AsL + RHPO, and AcL + AsL + RHPO.

RHPO significantly increased testicular MDA level compared to the control group (18.42 ± 2.26 vs. 14.35 ± 2.02; *p*˂0.01). Administration of AcL with RHPO (16.81 ± 2.03) decreased the MDA level but was not significant. While administration of AsL (15.62 ± 2.06) and a combination of AcL plus AsL (15.32 ± 1.36) with RHPO significantly decrease the MDA level versus RHPO (18.42 ± 2.26; *p*˂0.05). Administration of AcL, AsL, FPO, and SHPO failed to substantially affect testicular lipid peroxidation compared to the control group (Fig. 3).

**Fig. 4:**
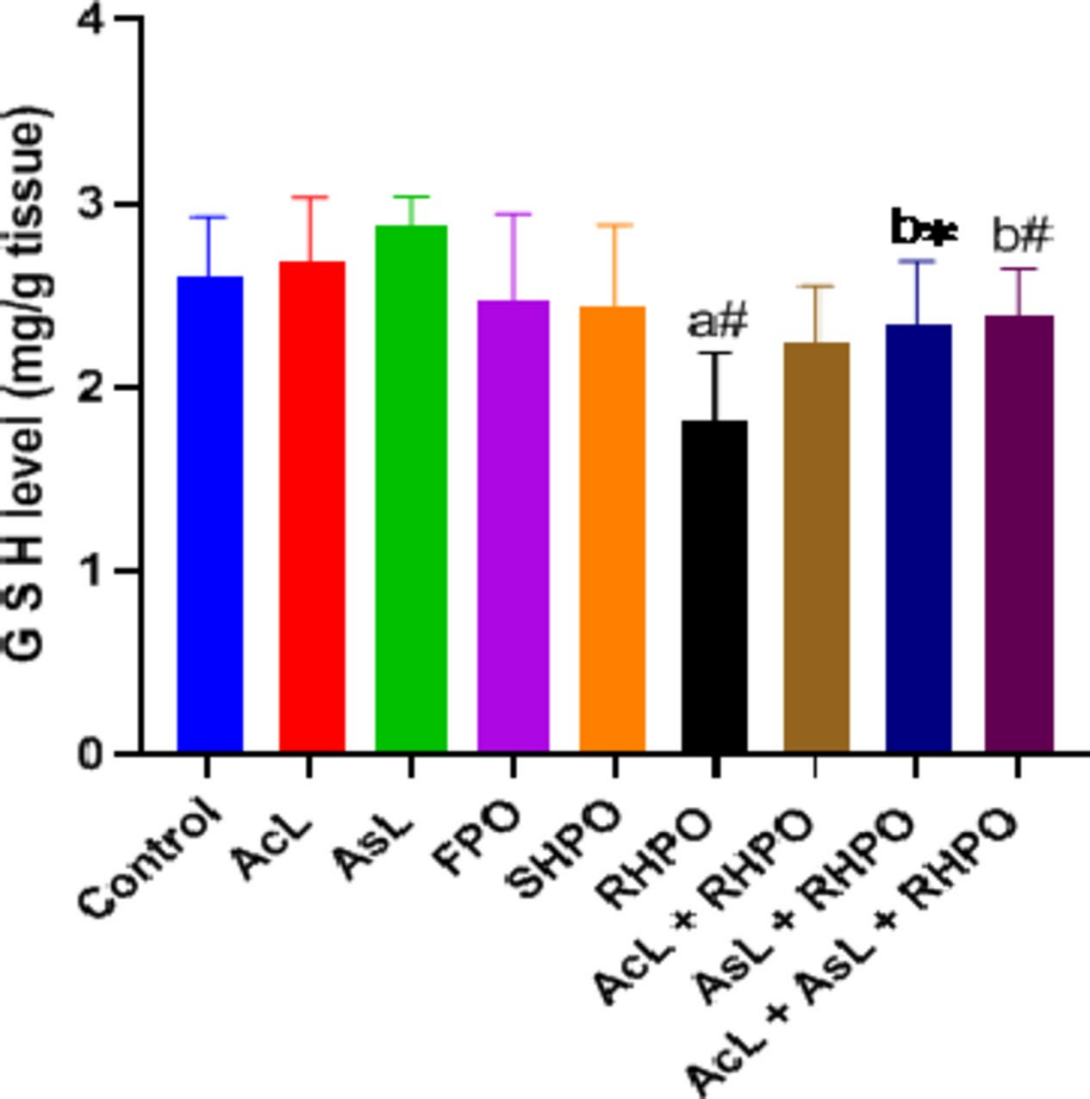
Effects of AcL and AsL extracts on the amount of testicular glutathione in rats given RHPO. With *n* = 8 in each group, the bars represent means ± SD. One-way ANOVA was used to analyze the data, followed by Turkey’s post hoc test. *p˂0.05 and #*p*˂0.01. a = control vs. AcL, AsL, FPO, SHPO, and RHPO. b = RHPO vs. AcL + RHPO, AsL + RHPO, and AcL + AsL + RHPO.

**Fig. 5:**
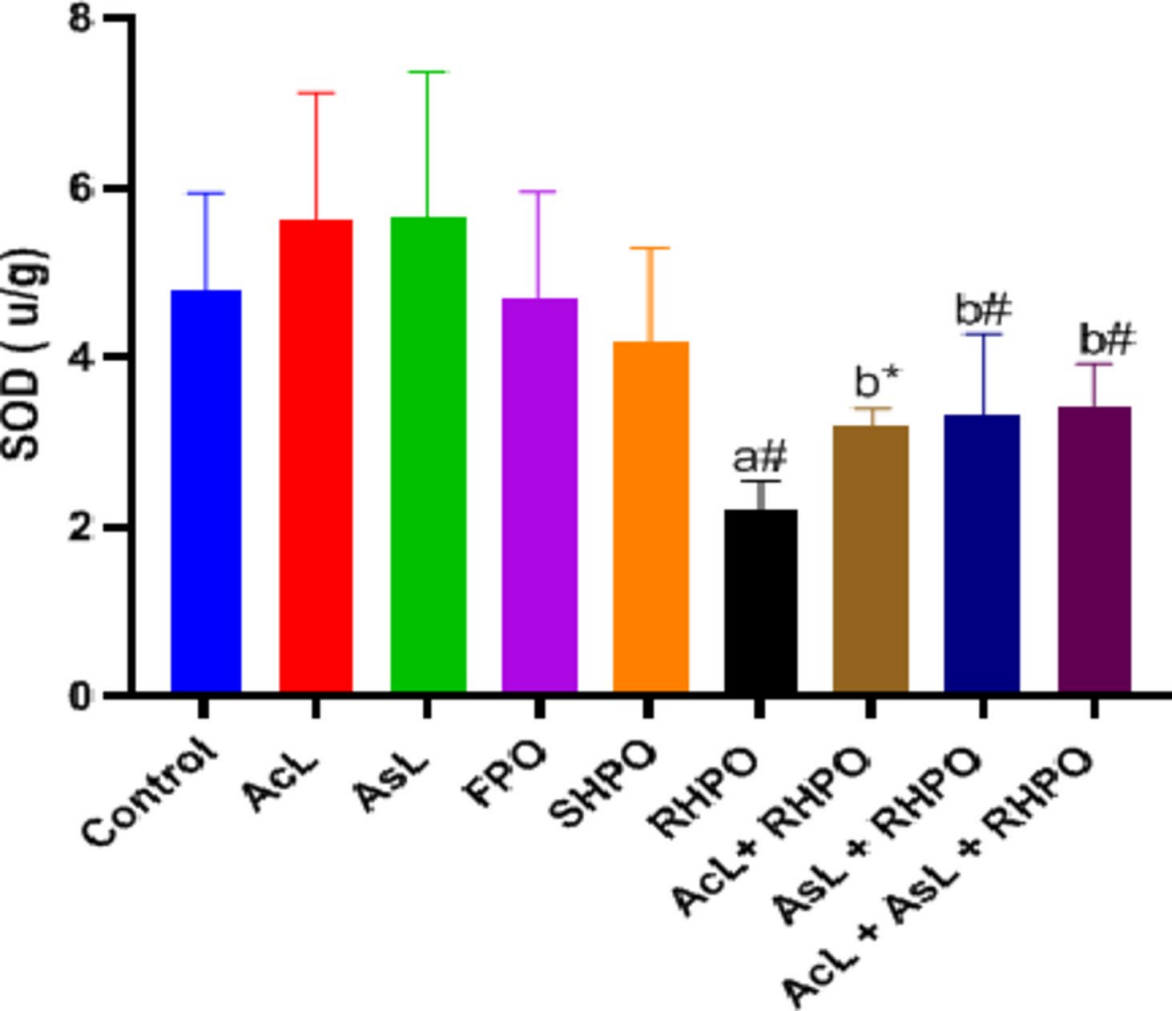
Effects of AcL and AsL extracts on the amount of testicular superoxide dismutase in rats given RHPO. With *n* = 8 in each group, the bars represent means ± SD. One-way ANOVA was used to analyze the data, followed by Turkey’s post hoc test. *p˂0.05 and #*p*˂0.01. a = control vs. AcL, AsL, FPO, SHPO, and RHPO. b = RHPO vs. AcL + RHPO, AsL + RHPO, and AcL + AsL + RHPO.

**Fig. 6:**
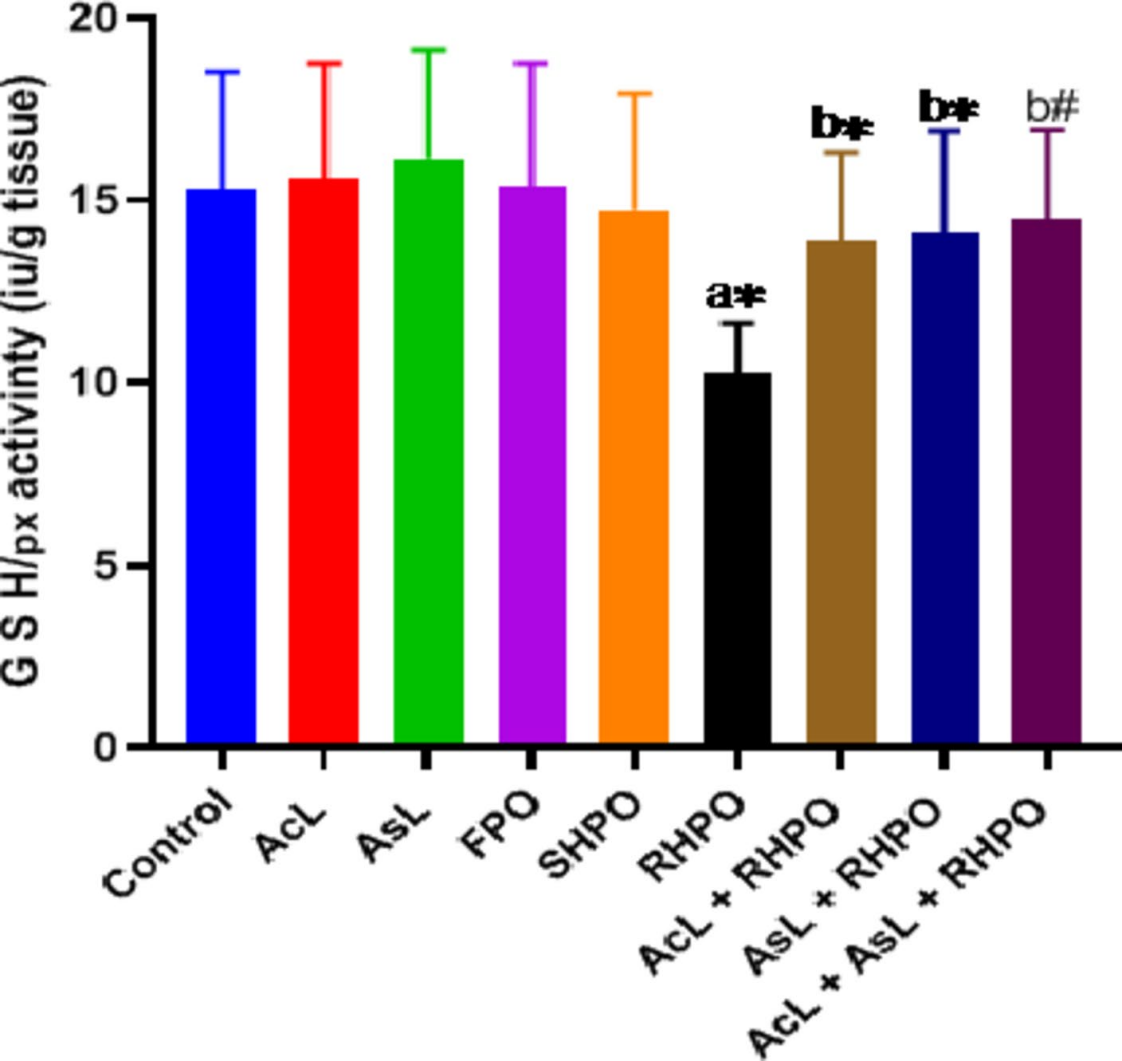
Effects of AcL and AsL extracts on the amount of testicular glutathione peroxidase in rats given RHPO. With *n* = 8 in each group, the bars represent means ± SD. One-way ANOVA was used to analyze the data, followed by Turkey’s post hoc test. *p˂0.05 and #*p*˂0.01. a = control vs. AcL, AsL, FPO, SHPO, and RHPO. b = RHPO vs. AcL + RHPO, AsL + RHPO, and AcL + AsL + RHPO.

Administration of RHPO significantly depleted testicular GSH levels compared to a control group (1.81 ± 0.36 vs. 2.59 ± 0.32; *p*˂0.01). Administration of AcL with RHPO (2.23 ± 0.31) increased GSH level but not significantly, while administration of AsL (2.34 ± 0.34) and AcL plus AsL (2.38 ± 0.25) with RHPO significantly increased GSH level versus RHPO (1.81 ± 0.36); *p*˂0.05 and *p*˂0.01 respectively), but no significant effect of AcL, AsL, FPO and SHPO on the testicular level of GSH when compared to control group. (Fig. 4). The rats exposed to RHPO had a significant reduction in activities of SOD (2.19 ± 0.34) in comparison with the control group (4.79 ± 1.13; *p*˂0.01). Administration of AcL (3.18 ± 0.21), AsL (3.3 ± 0.96), and AcL plus AsL (3.42 ± 0.48) with RHPO significantly increased the activities of SOD when compared with RHPO group (2.19 ± 0.34); *p*˂0.05, *p*˂0.01 and *p*˂0.01 respectively), while no significant changes in the testicular SOD level caused by AcL, AsL, FPO and SHPO versus control. (Fig. 5). RHPO significantly reduced GSH-Px testicular level compared to the control group (10.25 ± 1.37 vs.15.28 ± 3.23; *p*˂0.05). Administration of AcL (13.87 ± 2.43), AsL (14.11 ± 2.77), and AcL plus AsL (14.46 ± 2.46) with RHPO significantly increase the activities of GSH-Px level against RHPO group (10.25 ± 1.37); *p*˂0.05, *p*˂0.05 and *p*˂0.01 respectively. AcL, AsL, FPO, and SHPO failed to significantly affect the testicular level of GSH-Px against the control group (Fig. 6).

#### Serum level of testosterone

**Fig. 7:**
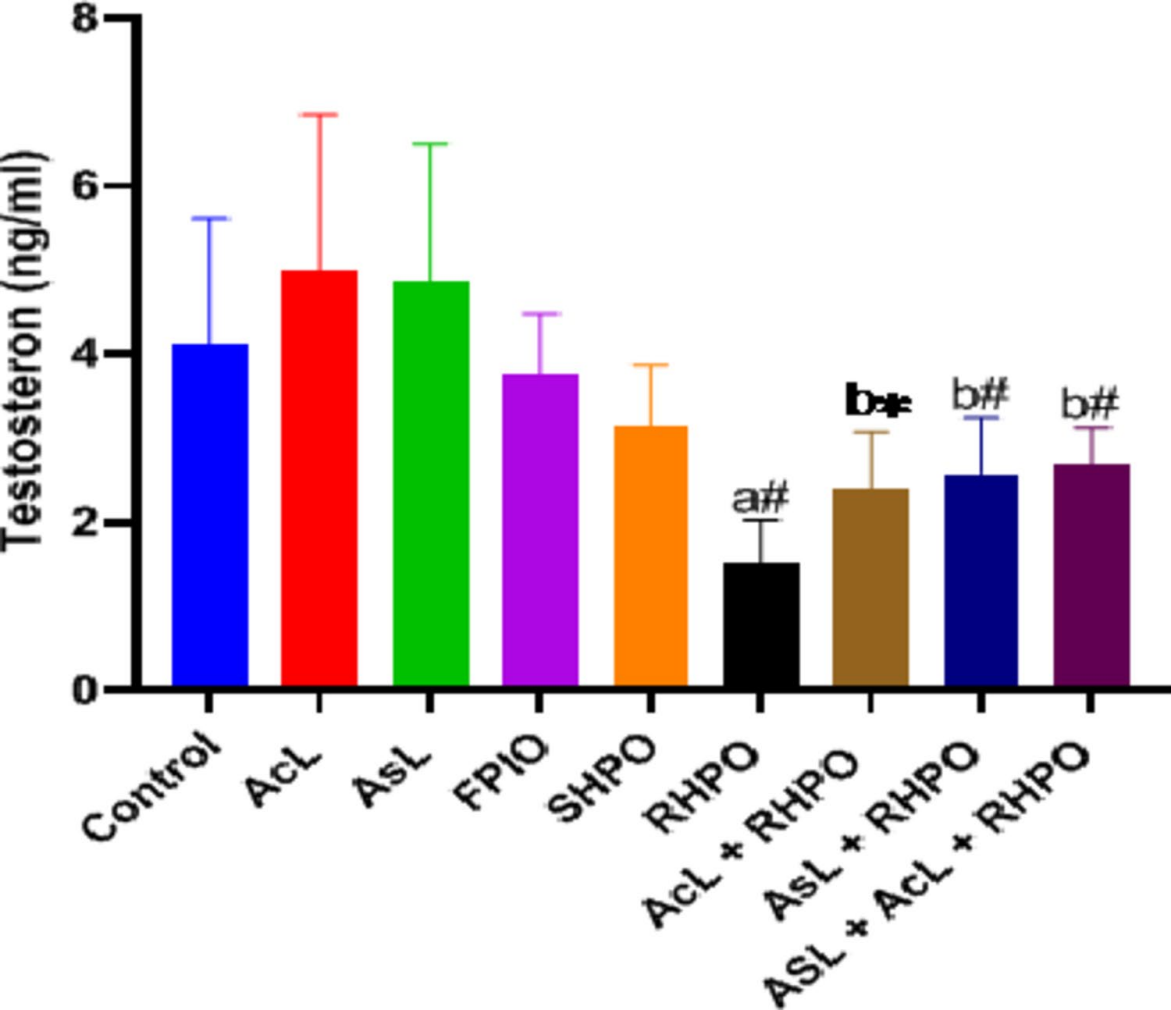
Effects of AcL and AsL extracts on the serum level of testosterone in rats given RHPO. With *n* = 8 in each group, the bars represent means ± SD. One-way ANOVA was used to analyze the data, followed by Turkey’s post hoc test. *p˂0.05 and #*p*˂0.01. a = control vs. AcL, AsL, FPO, SHPO, and RHPO. b = RHPO vs. AcL + RHPO, AsL + RHPO, and AcL + AsL + RHPO.

Figure 7 shows that compared to a control group, the administration of RHPO statistically reduced the amount of testosterone in the serum (1.52 ± 0.50 vs. 4.12 ± 1.48; *p*˂0.01). Whereas administration of AcL, AsL, and AcL plus AsL with RHPO significantly increase the testosterone level.

versus RHPO group (16.81 ± 2.03, 15.62 ± 2.06 and 15.32 ± 1.36 vs. 18.42 ± 2.26); *p*˂0.05, *p*˂0.01 and *p*˂0.01 respectively). There was no significant effect of AcL, AsL, FPO, and SHPO on the serum level of testosterone when compared to the control group.

### Sperm parameters

**Fig. 8:**
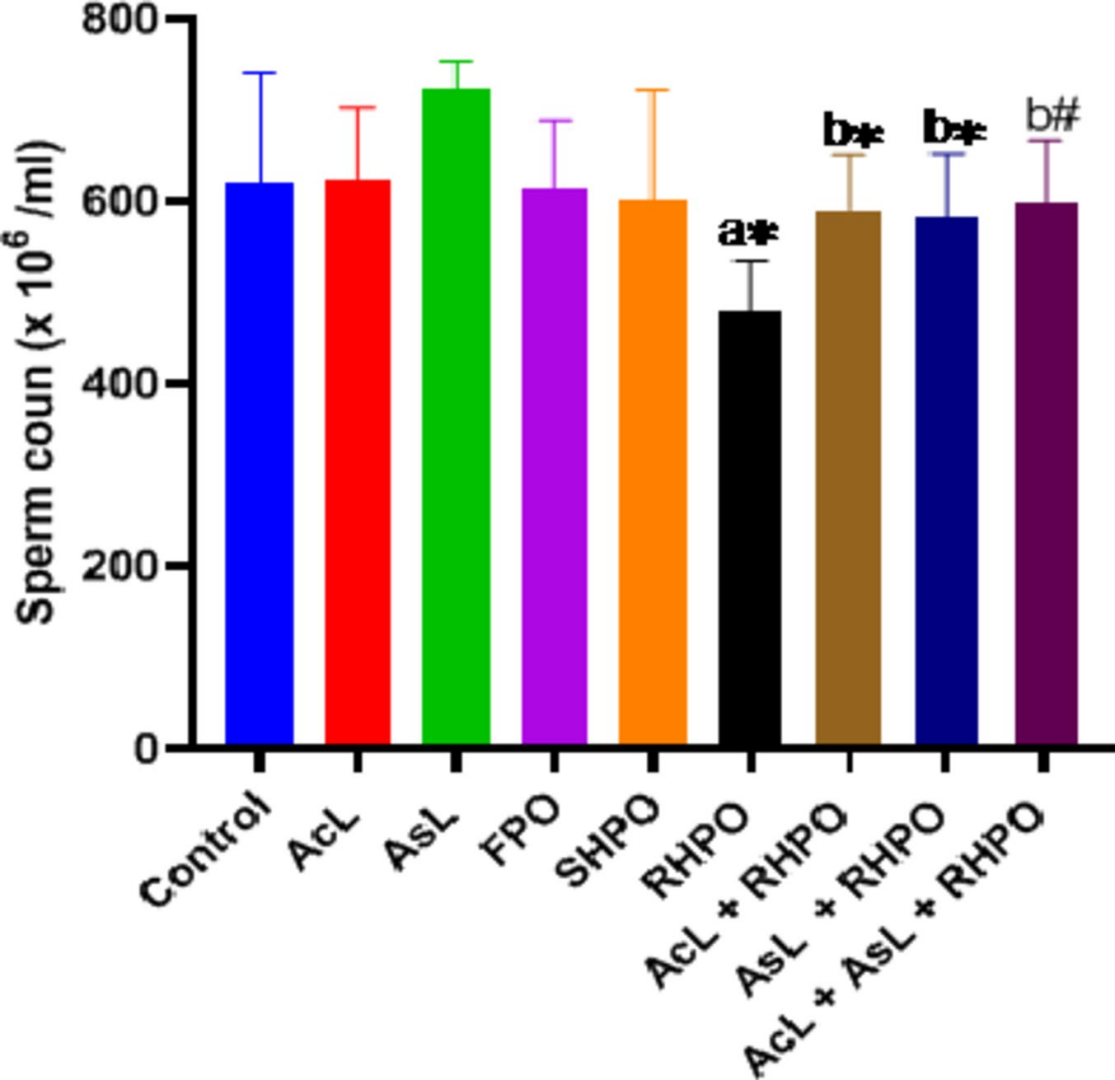
Effects of AcL and AsL extracts on the epididymal sperm count in rats given RHPO. With *n* = 8 in each group, the bars represent means ± SD. One-way ANOVA was used to analyze the data, followed by Turkey’s post hoc test. *p˂0.05 and #*p*˂0.01. a = control vs. AcL, AsL, FPO, SHPO, and RHPO. b = RHPO vs. AcL + RHPO, AsL + RHPO, and AcL + AsL + RHPO.

**Fig. 9:**
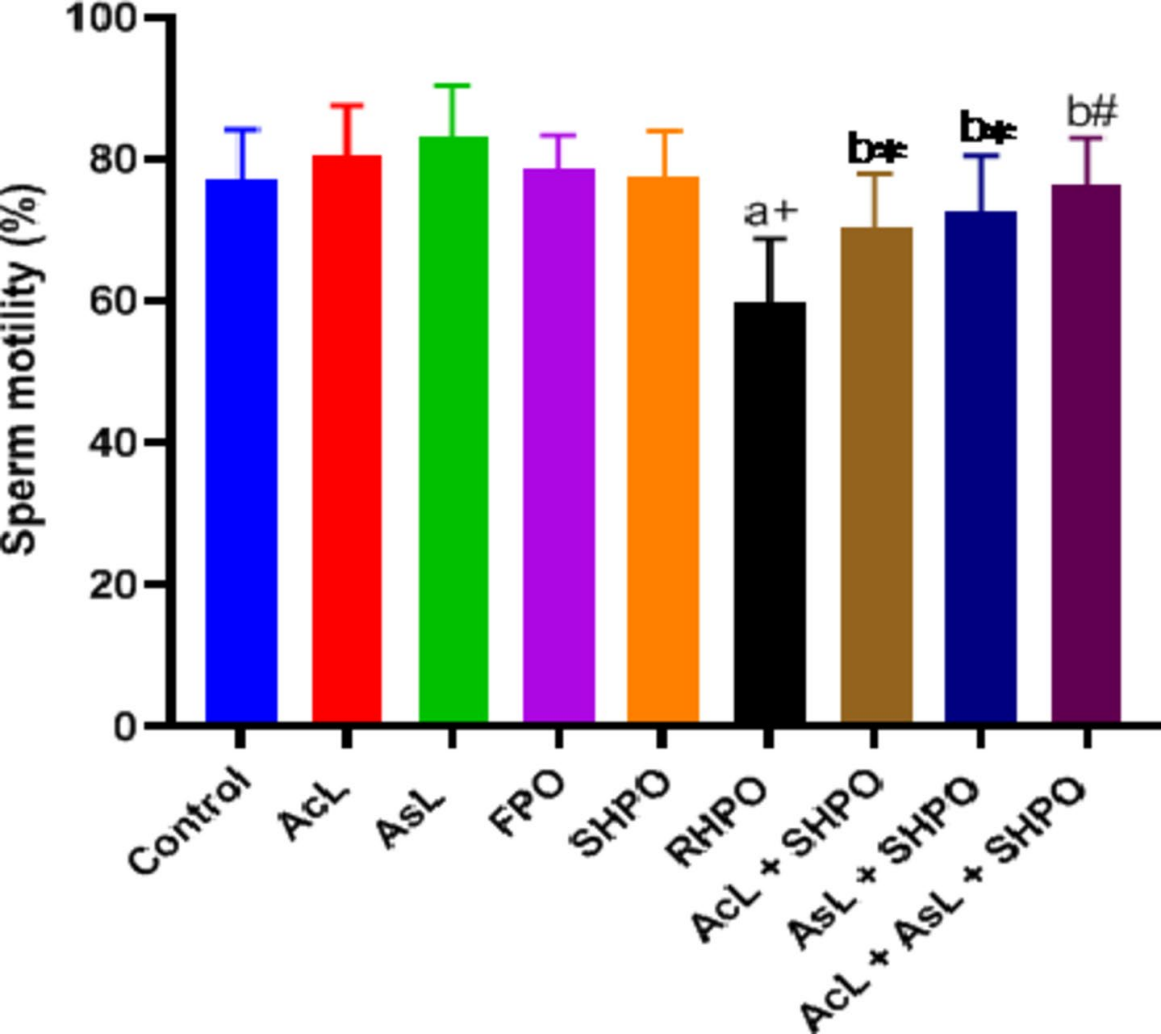
Effects of AcL and AsL extracts on epididymal sperm motility in rats given RHPO. With *n* = 8 in each group, the bars represent means ± SD. One-way ANOVA was used to analyze the data, followed by Turkey’s post hoc test. *p˂0.05, #*p*˂0.01 and + *p*˂0.001. a = control vs. AcL, AsL, FPO, SHPO, and RHPO. b = RHPO vs. AcL + RHPO, AsL + RHPO, and AcL + AsL + RHPO.

**Fig. 10:**
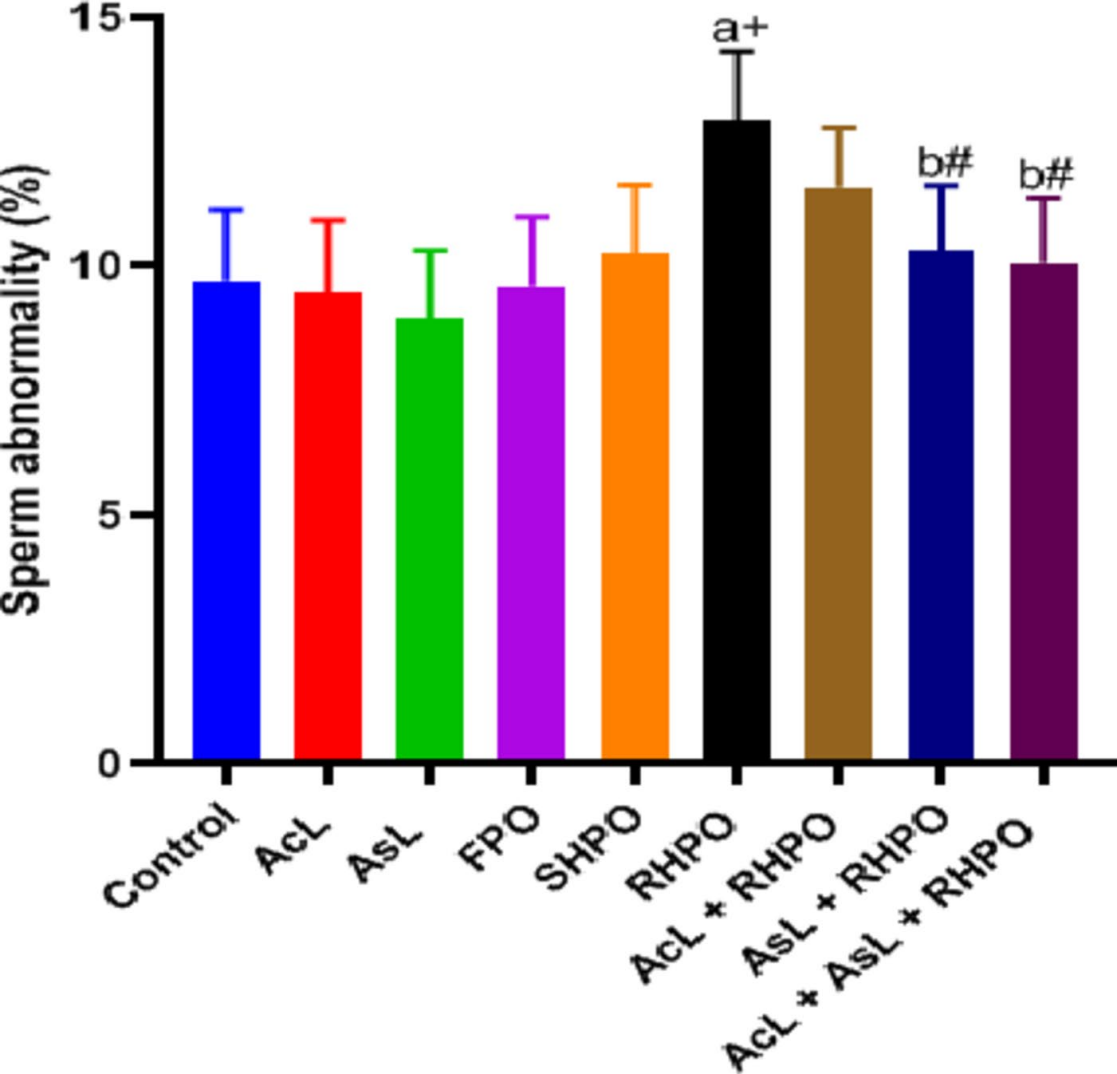
Effects of AcL and AsL extracts on abnormal sperm rate in rats given RHPO. With *n* = 8 in each group, the bars represent means ± SD. One-way ANOVA was used to analyze the data, followed by Turkey’s post hoc test: *p˂0.05, #*p*˂0.01 and + *p*˂0.001. a = control vs. AcL, AsL, FPO, SHPO, and RHPO. b = RHPO vs. AcL + RHPO, AsL + RHPO, and AcL + AsL + RHPO.

Figures 8, 9 and 10 display the epididymal sperm count, sperm motility, and aberrant sperm rate, respectively. Significant decreases in the number of epididymal sperm were seen when compared to the control group (480.35 ± 54.7 10^6 ^vs. 620.57 ± 120.36 10^6^; *p*˂0.05), sperm progress motility (59.84 ± 9% vs. 77.14 ± 7.03%; *p*˂0.001) with chronic administration of RHPO. Whereas administration of AcL, AsL and combination of them with RHPO when compared with RHPO group increase sperm count (590.57 ± 60.61 10^6^, 583.85 ± 68.31 10^6^ and 598 ± 68.9 10^6^ vs. 480.35 ± 54.7 10^6^; *p*˂0.05, *p*˂0.05 and *p*˂0.01 respectively), motility (70.42 ± 7.52%, 72.71 ± 7.8% and 76.42 ± 6.6% vs. 59.84 ± 9%; *p*˂0.05, *p*˂0.05 and *p*˂0.01 respectively).

Fig 10. shows that administration of RHPO significantly increases abnormal sperm rate compared to control (12.94 ± 1.36% vs. 9.7 ± 1.43%; *p*˂0.001). At the same time, administration of AcL with RHPO (11.58 ± 1.18%) decreased the abnormal rate but did not significantly, while AsL (10.3 ± 1.32%) and a combination of them (10.07 ± 1.29%) with RHPO decreased abnormal rate versus RHPO group (12.94 ± 1.36%); *p*˂0.01 and *p*˂0.01 respectively).

**Fig. 11:**
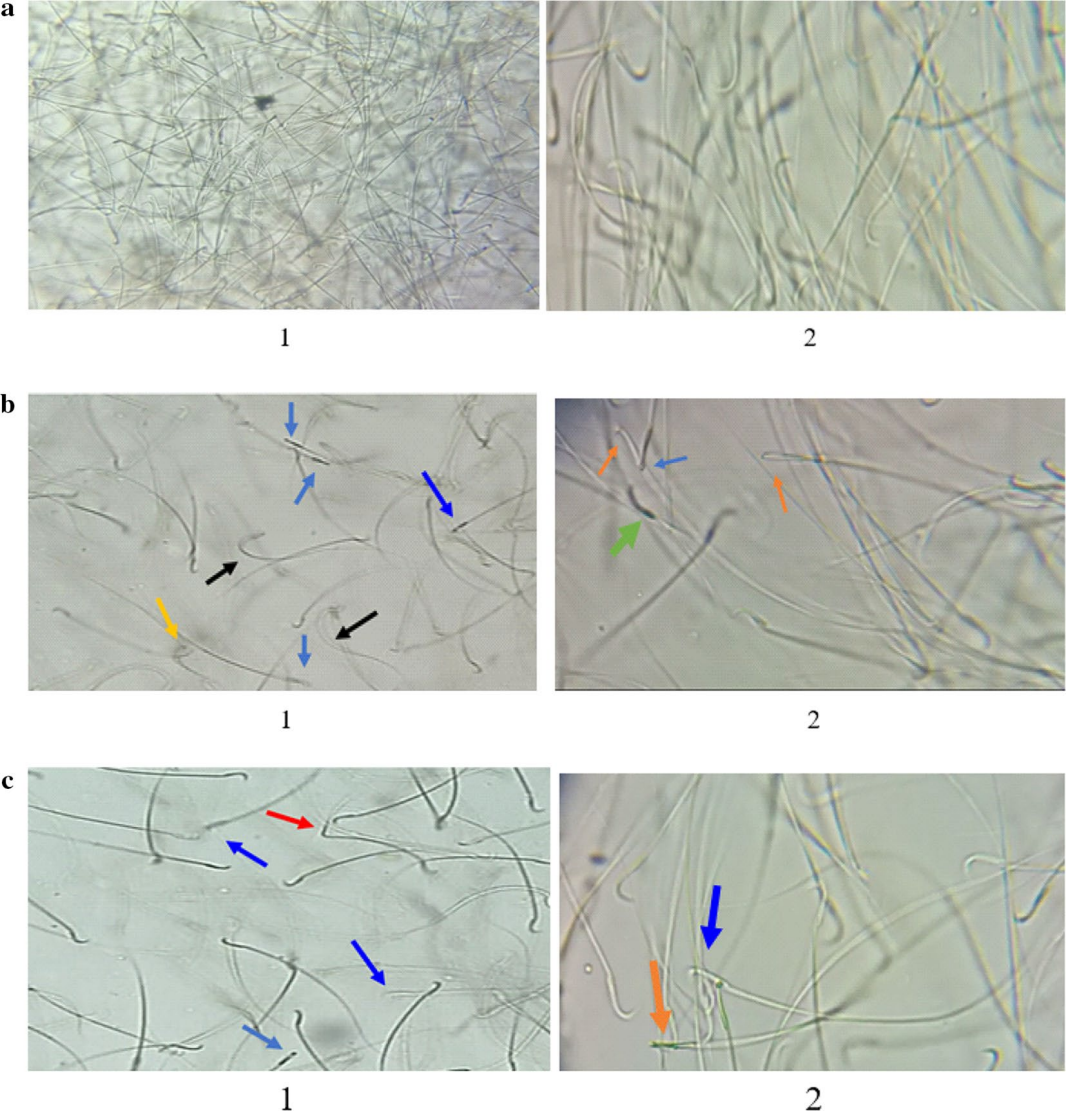

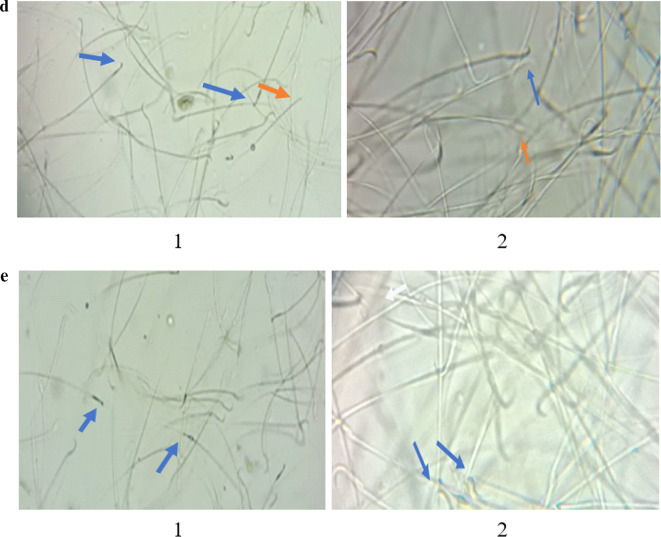
(**a**) Photomicrograph of control group shows normal number and morphologies of sperm with few abnormal forms (x 100). (**b**) **(1 & 2)**: Photomicrographs of the RHPO group show a decreased number and increased abnormal sperm morphologies compared to the control: straight head (blue arrow), coiled tail (yellow arrow), pent tail (red arrow), curved body (black arrow), and detached head (green arrow) (x 100). (**c**) **(1 & 2)**: Photomicrographs of acL with the RHPO group show an increased number and decreased abnormal sperm morphologies compared to the RHPO group: Straight head (blue arrow), absent head (brown arrow), and pent tail (red arrow) (x 100). (**d**) **(1 & 2)**: Photomicrographs of AsL with the RHPO group show an increased number and decreased abnormal sperm morphologies compared to the RHPO group: straight head (blue arrow), absent head (brown arrow) (x 100). (**e**) **(1 & 2)**: Photomicrographs of AcL plus AsL with the RHPO group showed an increased number and decreased abnormal sperm morphologies compared to the RHPO group: straight head (blue arrow) (x100).

 Fig. 11a shows a normal number and sperm morphologies of the control group.

Figure 11b (1&2**)** shows a decreased number and increased abnormal sperm morphologies in the RHPO group compared to control.

Fig. 11c,d, and e(1&2) show an increased number and decreased abnormal sperm morphologies of AcL with RHPO, AsL with RHPO, and AcL + AsL with RHPO as compared with RHPO group.

### Histopathological examination

**Figs. 12:**
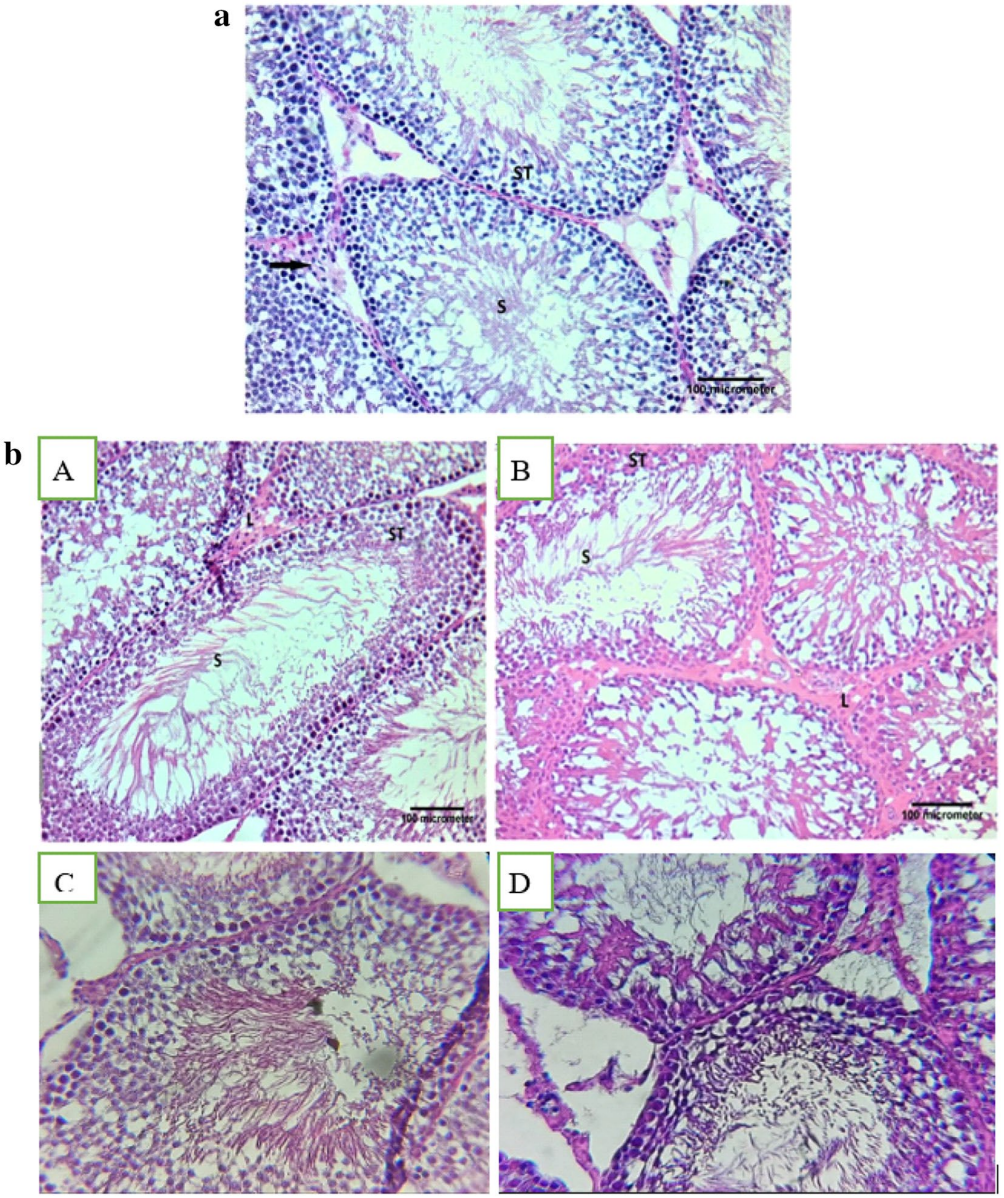

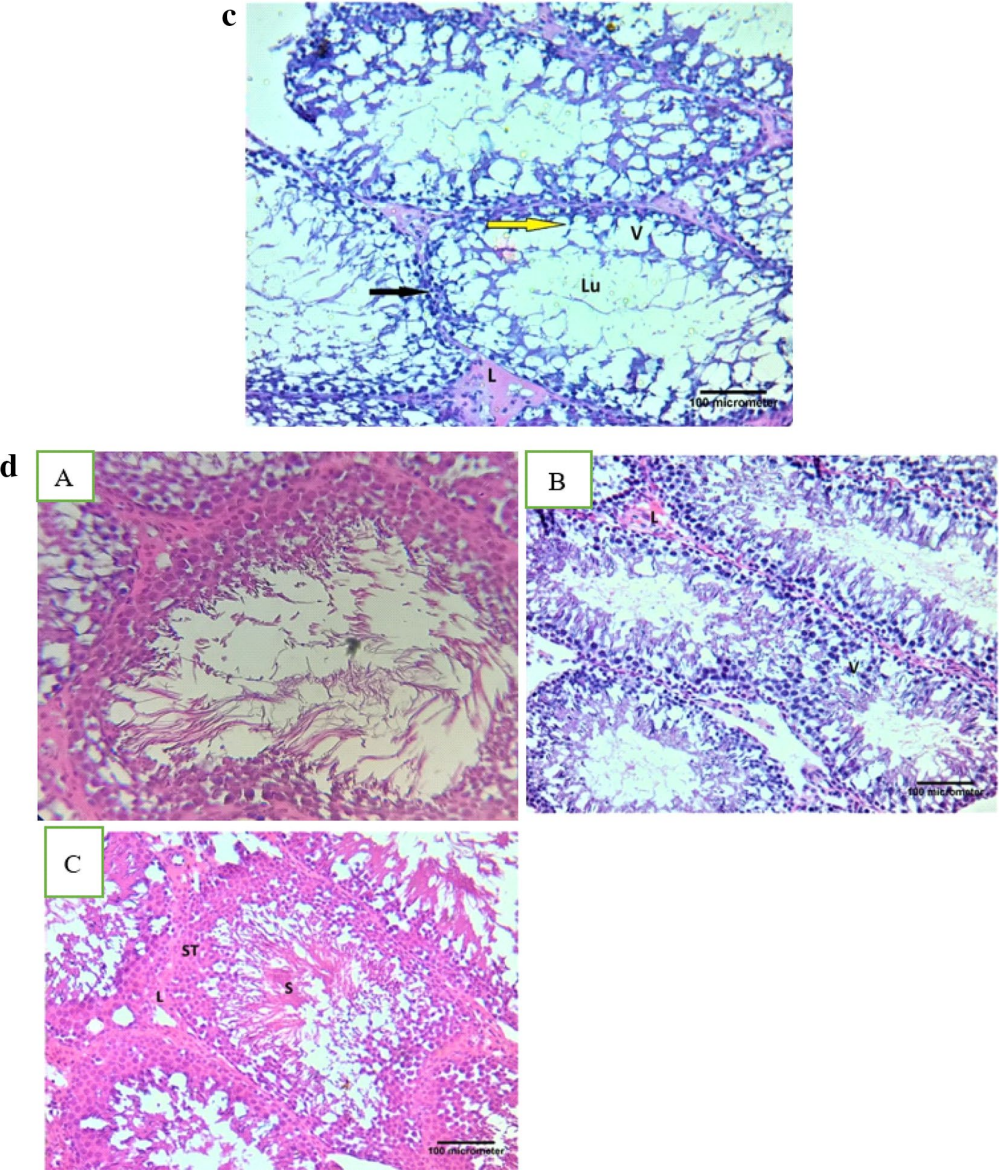
(**a**) Photomicrograph of the control group’s testicular slice demonstrating the closely packed, primarily spherical seminiferous tubules (ST) and the Leydig cell-containing interstitial tissue (arrow) between them. The lumen of the seminiferous tubule contains the sperms (S) (H&E X 200, scale bar = 100uµm). (**b**): Photomicrograph of testicular section of AcL group (A), AsL group (B), FPO group (C), and SHPO group (D) showing the same histological picture as the control group (H&E X 200, scale bar = 100uµm). (**c**): Photomicrograph of the RHPO group’s testicular section demonstrating the disordered and apparent reduction in spermatogenic cells. These cells feature pyknotic, tiny, darkly pigmented nuclei (yellow arrow) and vacuolated cytoplasm. The tubules have open areas and vacuolated portions (V). No sperm is visible in the tubules’ wide lumen (Lu) despite their size. The interstitial cells of Leydig (L) seem to be less in number, and the basement membrane of the seminiferous tubules seems to be broken and discontinuous (black arrow) (H&E X 200, scale bar = 100uµm). (**d**): Photomicrograph of testicular section of AcL + RHPO group (A), AsL + RHPO group (B), and AcL + AsL + RHPO group (C) demonstrates that the testis’s histological structure is almost identical to that of the control group, but vacuolated areas (V) can be detected within the tubules in AsL + RHPO group (B). The interstitial cells of the Leydig (L) are increased in number in AsL + RHPO group (B) and AcL + AsL + RHPO group (C) (H&E X 200, scale bar = 100uµm).

Sections from testes of rats of control, AcL, AsL, FPO, SHPO, RHPO, AcL with RHPO, AsL with RHPO, and AcL plus AsL with RHPO groups are shown in Fig. 12. Seminiferous tubules of a control group can be seen to be closely packed, typically spherical, with tight and organized of spermatogenic cells which consist of spermatogonia with small dark nuclei, primary spermatocytes with large vesicular nuclei and spermatids. Within the lumen of the seminiferous tubule, the sperms are visible. Interstitial tissue between seminiferous tubules containing blood capillaries and clusters of Leydig cells (Fig. 12a). AcL, AsL, FPO, and SHPO groups displayed the same histological picture as the control group (Figs. 12b).

The RHPO group’s testicular section demonstrates the disordered and apparent reduction in spermatogenic cells. These cells feature pyknotic, tiny, darkly pigmented nuclei and vacuolated cytoplasm. The tubules have open areas and vacuolated portions. No sperm is visible in the tubules’ wide lumen despite their size. The interstitial cells of Leydig seem to be less in number, and the seminiferous tubules’ basement membrane seems broken and discontinuous (Fig. 12c).

Figs. 12d. Photomicrograph of testicular section of AcL with RHPO, AsL with RHPO and AcL plus AsL with RHPO demonstrating that the testis’s histological structure is almost identical to that of the control group, but the (AsL-treated group) has tubules vacuolated regions. The number of Leydig interstitial cells appears to have grown in AsL and AcL + AsL-treated groups).

## Discussion

Refined palm olein oil is preferred in deep frying street food because of its low cost compared to other oils without knowing the harmful health effects of repeated heating. However, without knowing their beneficial role, ACL and ASL are basic constituents in most street foods, including falafel. To our knowledge, no literature report is available on the effect of ACL and ASL extracts on the testes of rats exposed to chronic administration of RHPO.

Consequently, the current investigation is unique. Rats’ testicles were rendered toxic by deep-frying street falafel dipped in palm oil, as our earlier study^14^ showed. The primary pathophysiology of testicle anomalies may be oxidative damage caused by producing reactive oxygen species (ROS) and free radicals. In this investigation, we assessed the impact of ACL and ASL extracts on the testes of rats given a chronic dose of hot, palm oil-fried street falafel regularly.

Our results in the current study indicated that administration of RHPO caused a tremendous increase in the level of testicular lipid peroxidation (as indicated by the significant increase in MDA content) and a considerable decrease in the levels of testicular GSH, SOD, and GSH-Px. No significant changes occurred by SHPO. These results are similar to that of our previous study^14^.

Recently, Zakaria et al.^26^ mentioned that multiple-heated cooking oil induced hepato-renal damage through increased oxidative stress, tumor necrosis factor-alpha, Bax, and fibrosis of liver tissues; in addition, multiple-heated cooking oil enhanced the hepatic expression of cell cycle markers p53, p21, cyclin D, and proliferating cell nuclear antigen (PCNA).

In agreement with our results, Wang et al.^27^ reported that a high-fat diet-induced testicular damage, oxidative stress, and apoptotic germ cell death. In agreement with our findings, Kamisah et al.^28^ noted that up to two heating of palm oil, the peroxide values of the oil were still below the maximum limit of the peroxide value. They recommended that palm oil should not be heated more than two times for safe consumption.

Lipid peroxidation results from changes in the redox state brought on by excess free radicals. Despite being an unimpeded natural process, lipid peroxidation is essential in fundamental deteriorative mechanisms, such as nucleic acid mutagenesis, enzyme degradation, and cell injury^29^. GSH protects vital thiol groups from oxidation, reacts directly with ROS and electrophilic metabolites, and acts as a substrate for several enzymes, including GSH-Px^30^.

Changing GSH’s redox state can increase oxidative stress and tissue damage and compromise cells’ ability to defend against harmful substances^31^.

Our discovery of GSH depletion could result from increased use to combat lipid peroxidative products and ROS. To avoid oxidative stress, SOD and GSH-Px are vital components of the cellular antioxidant defense mechanism. The substantial reduction in SOD and GSH-Px activity in the RHPO-treated group could be explained by ROS’s extensive oxidative alteration of enzyme proteins and biomembrane lipids, which is demonstrated by elevated lipid peroxidation.

These antioxidant enzymes’ primary function is to quickly convert superoxide radicals into hydrogen peroxide (H_2_O_2_) in the presence of SOD. Due to increased mitochondrial oxygen consumption during spermatogenesis in the testis^32^ or phagocytosis of germ cell debris in the testis by testicular somatic cells, which produces superoxide radical byproducts, superoxide radical is the most easily generated free radical^33^.

In addition, the Fenton reaction can change the H_2_O_2_ produced in the presence of Fe2 + into the deadly hydroxyl radical (*OH), another free radical^34^. As a result, the H_2_O_2_ produced is quickly removed from the cell to stop DNA, lipids, and proteins from suffering oxidative damage. By using reduced glutathione (GSH) as the electron donor and H2O as the product, GSH-Px can eliminate H_2_O_2_. Glutathione, a significant cellular antioxidant, prevents oxidative cell damage. While reducing H2O2 and other peroxides, GSH directly interacts with ROS and serves as a cofactor for GSH-Px^35^.

There are various ways to prevent oxidative stress and lessen the harmful effects of reactive oxygen species. One of these processes is the antioxidant system, which scavenges free radicals. As a result, eating vegetables high in antioxidants may help lessen the oxidative stress linked to RHPO testicular toxicity. As demonstrated by lower levels of lipid peroxidation and improved antioxidant defense status, ACL and ASL extracts in this study partially shield rats against RHPO-induced testicular damage. Flavonoids (quercetin, allixin, anthocyanins, and kaempferol) and cysteine-containing bioactive chemicals (alkyl cysteine sulphoxide) are present in ACL and ASL and are recognized to have antioxidant properties^36,37^.

However, because palm oil contains a lot of vitamin E, a potent lipophilic antioxidant, and falafel dough contains ACL and ASL, heating the oil repeatedly may significantly impact their actions.

The current study found that long-term RHPO treatment reduced the number of epididymal sperm, suppressed sperm motility, and significantly increased sperm abnormalities. Furthermore, the current study’s findings concur with our earlier investigation^14^.

One explanation is that RHPO may directly harm sperm quality. Another theory is that H_2_O_2_, one of the byproducts of lipid peroxidation, may permeate the membrane and impact the sperms’ essential enzymes, reducing their motility. Increased sperm membrane lipid peroxidation has been demonstrated to impair sperm progress motility, increase the percentage of total sperm abnormalities, and cause a significant decline in sperm fertilizing potential, according to Sharma and ArASLwal^38^.

Additionally, GSH deficiency has been linked to sperm mid-piece instability, which results in impaired motility, according to Hansen and Deguchi^39^. Their antioxidant defense capability significantly impacts how much oxidative damage happens in the testis and sperm^40^. The process of spermatogenesis is a very dynamic one. The germinal epithelium must consume mitochondrial oxygen at high rates due to the high cell division rates inherent in this process. Leydig cell steroidogenesis and spermatogenesis are susceptible to oxidative stress. In spermatozoa, ROS can disrupt mitochondrial activity and RNA synthesis^41,42^.

According to the current study, administering ACL and ASL extracts may help with testicular histological abnormalities brought on by RHPO and sperm parameters. Numerous antioxidants, including quercetin, selenium, glutathione, and vitamins, may be responsible for these benefits. According to Samson et al.^43^, the protective effects of ACL and ASL extracts may be caused by their high antioxidant content, which includes vitamins A, B, and C, selenium, glutathione, and flavonoids (quercetin and isorhamnetin).

The present study’s results are consistent with those of Marefati et al.^44^ and Nikzad et al.^45^, who found that consuming extracts of ACL and ASL significantly reduced oxidative stress in the rat testes. Furthermore, it was suggested by Nikravesh et al.^46^ that mice’s testicular histological alterations might be lessened by consuming ACL.

Rats treated with RHPO had reduced testosterone levels, according to the study’s findings. Increases in oxidative stress are thought to cause testicular Leydig cells to secrete less testosterone because testicular tissue is highly vulnerable to free radicals^47^. Additionally, it is advised to use antioxidant supplements to improve spermatogenesis and fertility, reduce oxidative stress, and neutralize free radicals [35]. Because of their antioxidant qualities, ACL and ASL make excellent candidates for this use. The histological investigation corroborated the biochemical findings.

## Conclusion

The study highlighted that the chronic consumption of repeatedly heated palm olein oil-fried street falafel is associated with a significant reduction of the testicular antioxidant enzymatic system and induced testicular oxidative stress and tissue damage in rats compared to single heated palm olein oil. The co-administration of ACL and ASL extracts reduced RHPO-induced oxidative stress by decreasing lipid peroxidation and activating antioxidant enzymes in the testes, therefore ameliorating RHPO-induced testicular toxicity in rats. The study demonstrated that SHPO is the safe dose. Further studies are needed to clarify the molecular mechanisms responsible for oxidative stress induced by RHPO and the ameliorating effect of ACL and ASL extracts on testes and sperm parameters. Also, further studies are required to evaluate the quality of repeatedly heated edible oils used for preparing fast Egyptian street food and strict observation by health government authorities. Animal and human studies are also needed to evaluate the formation of DNA aberration associated with RHPO consumption in a model that mimics the human situation and considers the reasons for the increasing incidences of cancer and cardiovascular diseases in low and middle-income countries.

## Data Availability

“Data is provided within the manuscript”.
